# Direct Selective Epitaxy of 2D Sb_2_Te_3_ onto Monolayer WS_2_ for Vertical p–n Heterojunction Photodetectors

**DOI:** 10.3390/nano14100884

**Published:** 2024-05-19

**Authors:** Baojun Pan, Zhenjun Dou, Mingming Su, Ya Li, Jialing Wu, Wanwan Chang, Peijian Wang, Lijie Zhang, Lei Zhao, Mei Zhao, Sui-Dong Wang

**Affiliations:** 1 Macao Institute of Materials Science and Engineering (MIMSE), MUST-SUDA Joint Research Center for Advanced Functional Materials, Macau University of Science and Technology, Taipa, Macao 999078, China; 2 Key Laboratory of Carbon Materials of Zhejiang Province, Institute of New Materials & Industry Technology, College of Chemistry and Materials Engineering, Wenzhou University, Wenzhou 325035, China; 3 School of Electronic Engineering, Lanzhou City University, Lanzhou 730070, China

**Keywords:** substrate-selective growth, Sb_2_Te_3_/WS_2_ vertical heterojunction, p–n heterojunction, chemical vapor deposition, photodetector

## Abstract

Two-dimensional transition metal dichalcogenides (2D-TMDs) possess appropriate bandgaps and interact via van der Waals (vdW) forces between layers, effectively overcoming lattice compatibility challenges inherent in traditional heterojunctions. This property facilitates the creation of heterojunctions with customizable bandgap alignments. However, the prevailing method for creating heterojunctions with 2D-TMDs relies on the low-efficiency technique of mechanical exfoliation. Sb_2_Te_3_, recognized as a notable p-type semiconductor, emerges as a versatile component for constructing diverse vertical p–n heterostructures with 2D-TMDs. This study presents the successful large-scale deposition of 2D Sb_2_Te_3_ onto inert mica substrates, providing valuable insights into the integration of Sb_2_Te_3_ with 2D-TMDs to form heterostructures. Building upon this initial advancement, a precise epitaxial growth method for Sb_2_Te_3_ on pre-existing WS_2_ surfaces on SiO_2_/Si substrates is achieved through a two-step chemical vapor deposition process, resulting in the formation of Sb_2_Te_3_/WS_2_ heterojunctions. Finally, the development of 2D Sb_2_Te_3_/WS_2_ optoelectronic devices is accomplished, showing rapid response times, with a rise/decay time of 305 μs/503 μs, respectively.

## 1. Introduction

Two-dimensional materials are pivotal in advancing the miniaturization of electronic devices to enhance Moore’s Law, owing to their unique atomic-scale thickness [1,2]. The van der Waals interlayer interaction in these materials overcomes lattice-matching challenges faced in conventional heterojunctions [3], enabling a “Lego-like” stacking approach in layered heterostructures, which serves as an excellent research platform in optics [4], electronics [5], magnetism [6,7], and other fields [8].

Two-dimensional transition metal dichalcogenides (2D-TMDs) are distinguished among the materials investigated due to their layered structure, which includes appropriate bandgaps and chemical stability [9,10]. These characteristics render them highly suitable for optoelectronic device fabrication. In particular, the p–n heterojunction is a crucial structure in photodetectors, as it facilitates efficient separation of photogenerated electrons. Intrinsic two-dimensional transition metal chalcogenides are primarily n-type semiconductors, with p-type semiconductors being less common. For example, monolayer PtSe_2_ is a p-type semiconductor with an indirect band gap of 1.16 eV [11,12,13]. To achieve high-performance photodetectors, this work introduces Sb_2_Te_3_, a p-type semiconductor with a direct band gap of 0.33 eV [14,15], to construct p–n heterojunctions with n-type two-dimensional transition metal chalcogenides. Nevertheless, the current approach to building novel two-dimensional layered material heterostructures primarily relies on the repetitive and inefficient mechanical exfoliation process [16], which is not conducive to mass industrial production.

Considered a method with potential for large-scale, controllable fabrication of 2D-TMD [2], chemical vapor deposition (CVD) is compatible with existing semiconductor processes and can produce high-quality crystals. The synthesis of two-dimensional transition metal dichalcogenide heterostructures via CVD involves both one-step and two-step processes. For instance, Jiao et al. demonstrated a one-step procedure wherein they sulfurized WO_3−x_/MoO_3−x_ core-shell nanowires, resulting in vertically stacked MoS_2_/WS_2_ heterojunctions [17]. In contrast, a two-step CVD method, as employed by Duan et al., involves initial laser etching to create nucleation sites on large-area WSe_2_ surfaces, followed by epitaxial growth of VSe_2_ to produce arrayed VSe_2_/WSe_2_ vertical heterojunctions [18]. During the synthesis of two-dimensional transition metal chalcogenide heterostructures, the one-step method can easily lead to atomic intermixing within the heterostructures [19]. In contrast, the two-step method requires careful avoidance of surface contamination on the two-dimensional materials produced in the first step to prevent interference with the epitaxial growth of the two-dimensional transition metal chalcogenides in the second step [20,21].

To date, there have been no reports on the preparation of Sb_2_Te_3_/WS_2_ p–n heterojunctions using CVD. Therefore, this paper attempts to fabricate Sb_2_Te_3_/WS_2_ p–n heterojunctions using a two-step chemical vapor deposition method. Initially, to explore the preparation conditions for two-dimensional Sb_2_Te_3_, we achieved large-scale fabrication on inert mica substrates as a reference for constructing related Sb_2_Te_3_ heterostructures. Due to differences in surface migration barriers, van der Waals layered materials tend to selectively epitaxially grow on surfaces with lower migration barriers [22,23,24]. Therefore, by exploiting the relatively low migration barrier of Sb_2_Te_3_ on WS_2_ surfaces, this work adopts a two-step chemical vapor deposition strategy to selectively epitaxially grow Sb_2_Te_3_ on pre-prepared WS_2_ on SiO_2_/Si substrates, achieving the assembly of Sb_2_Te_3_/WS_2_ heterojunctions.

Lastly, the two-dimensional Sb_2_Te_3_/WS_2_ heterostructures were utilized in the assembly of photodetectors, achieving in rapid response times of a 302-microsecond rise time and 503-microsecond decay time. This research provides valuable insights into constructing heterojunctions involving Sb_2_Te_3_ and other two-dimensional layered materials, contributing to the advancement of high-performance photodetectors. Furthermore, given that two-dimensional materials with atomic-level thickness exhibit excellent mechanical flexibility [25] and high integration, they hold potential applications in industrial flexible robotic sensors.

## 2. Materials and Methods

### 2.1. Materials

Tungsten Trioxide Powder (WO_3_, 99.99%), and Antimony Trioxide Powder (Sb_2_Te_3_, 99.9%) were purchased from Alfa Aesar, Waltham, MA, USA. Fluorophlogopite Mica substrates were sourced from Taiyuan Fluorophlogopite Co., Ltd. in Changchun City, China. Chromium Metal (Cr, 99.99%) and Gold Metal (Au, 99.99%) were purchased from Beijing Zhongjin Yanxin Materials Technology Co., Ltd. in Beijing, China. 285 nm of SiO_2_/Si substrates were obtained from Hefei Kejin Material Co., Ltd. in Hefei, China.

### 2.2. Synthesis of 2D-Sb_2_Te_3_

To synthesize 2D-Sb_2_Te_3_, a quartz boat containing 50 mg of Sb_2_Te_3_ powder was placed at the center of a single-zone tube furnace (with a quartz tube diameter of 1.0 inch). A mica substrate was placed 5 cm away from the center of the boat. Prior to heating, the system underwent a 30-min purge with Ar gas; once heating commenced, the Ar flow rate was set to 50 standard cubic centimeters per minute (sccm). Subsequently, the temperature was increased at a rate of 25 °C/min up to 650 °C and maintained for 2 min. Ultimately, the quartz tube was swiftly cooled to room temperature, and the sample was extracted.

### 2.3. Synthesis of Monolayer WS_2_

To synthesize monolayer WS_2_, the 280 nm SiO_2_/Si underwent a sequential treatment involving ultrasonic in acetone, isopropanol, and deionized water, each for 30 min. Subsequent to the ultrasonic treatment, the SiO_2_/Si was dried using a nitrogen gun, annealed in a muffle furnace at 600 °C for a duration of four hours, allowed to cool naturally to room temperature, and then set aside. Following this, 2.5 mg of WO_3_ powder was precisely measured and uniformly dispersed on a custom-made graphite trough. A SiO_2_/Si substrate measuring 1.5 cm × 1.0 cm was delicately positioned 0.5 cm above the WO_3_ powder in the trough. Additionally, 300 mg of sulfur powder was weighed and placed in a quartz trough fitted with a magnetic pull rod. The graphite trough containing SiO_2_/Si substrate and the quartz boat containing sulfur powder were positioned at the center and outside the heating region of a one-inch tube furnace. The quartz tube was purged with argon for 30 min to eliminate air and moisture. The argon flow rate was adjusted to 40 sccm, and the temperature was gradually increased at a rate of 25 °C/min up to 900 °C. Upon reaching the specified temperature, the sulfur powder was shifted to an area close to 380 °C, underwent a 5-min reaction, followed by the removal of the sulfur powder, rapid cooling to room temperature, and retrieval of the sample.

### 2.4. Synthesis of 2D-Sb_2_Te_3_/WS_2_ Heterostructure

For the fabrication of the 2D-Sb_2_Te_3_/WS_2_ heterostructure, a precise amount of 50 mg Sb_2_Te_3_ powder was positioned within a quartz boat placed at the central temperature zone of a single-zone tube furnace (with a quartz tube diameter of 1.0 inch). Concurrently, a SiO_2_/Si substrate, coated with a monolayer of WS_2_ flakes, was strategically placed 5 cm away from the center of the quartz tube. Before initiating the heating process, the system underwent a thorough 30-min Ar purging, followed by the adjustment of the Ar flow rate to 50 sccm. The temperature was then steadily increased at a rate of 25 °C/min until reaching 650 °C, where it was held for 2 min. Ultimately, the quartz tube was cooled rapidly to ambient temperature, and the sample was extracted.

### 2.5. Device Fabrication and Testing

For the fabrication of optoelectronic devices, standard e-beam lithography and metal thermal evaporation techniques were used to define electrodes Cr/Au (10/50 nm). Optoelectronic measurements were conducted with the probe station CRX-6.5K from Lake Shore and the semiconductor parameter analyzer Keithley 4200 SCS (Cleveland, OH, USA) at room temperature under a 532 nm laser. Laser power density was collected with a powermeter (Thorlabs GmbH., PM 100D, Dachau, Germany).

### 2.6. Characterization

The morphology of the samples was examined using optical microscopy (OM, Carl Zeiss Microscopy GmbH, Jena, Germany) and scanning electron microscopy (SEM, Nova Nano SEM 200 FEI, Hillsboro, OR, USA). Their chemical compositions were assessed through Raman and photoluminescence (PL) spectra at room temperature. Raman spectra were obtained using a micro confocal Raman/PL spectrometer (Renishaw in Via, Gloucestershire, UK) with an excitation laser line operating at 532 nm. Flake thickness measurements were conducted using atomic force microscopy (AFM) with a Dimension Icon system from Bruker, San Diego, CA, USA. The crystallinity quality and chemical composition of the Sb_2_Te_3_ and Sb_2_Te_3_/WS_2_ flakes were investigated using transmission electron microscopy (TEM, JEOL 2100F, Tokyo, Japan) and X-ray photoelectron spectroscopy (XPS) analysis performed on a PHI 5000 VP III instrument, Woodbury, MN, USA.

### 2.7. Density Functional Theory (DFT) Calculations

Geometrical optimizations were performed using DFT with Perdew–Burke–Ernzerhof generalized gradient approximation (PBE-GGA) functional employing projector augmented wave (PAW) potentials, implemented in the Vienna Ab-initio Simulation Package (VASP). The band structure of monolayer WS_2_ and the band structure of 2D Sb_2_Te_3_ were calculated separately based on their respective structures. A kinetic cutoff energy of 450 eV was chosen for the planewave basis set. The valence electron configurations for W (5p^6^6s^2^5d^4^), S (3s^2^3p^4^), Sb (5s^2^5p^3^), Te (5s^2^5p^4^) were employed. The first Brillouin zone was characterized by a Γ-point-centered Monkhorst-Pack *k*-mesh with a grid configuration of 6 × 6 × 4. The energy convergence criterion was set at 1.0 × 10^−4^ eV, for both structural optimizations and self-consistent-field (SCF) iteration. The force components convergence criterion was set at −0.02 eV/Å, with the charge density symmetrization employed.

## 3. Results and Discussion

### 3.1. Morphological and Structural Characterization of WS_2_, Sb_2_Te_3_, and Sb_2_Te_3_/WS_2_ Heterojunction

This paper describes the fabrication process of Sb_2_Te_3_/WS_2_ heterostructures utilizing a two-step chemical vapor deposition method. Initially, we successfully prepared large-area monolayer WS_2_ on SiO_2_/Si substrate. Figure S1a shows an optical image of monolayer WS_2_ flakes uniformly distributed on SiO_2_/Si substrate. Additionally, a single WS_2_ flake was subjected to AFM, revealing a measured thickness of approximately 0.83 nm, consistent with reported monolayer WS_2_ thicknesses, as shown in Figure S1b [26]. Moreover, Raman spectroscopy was performed on the obtained WS_2_. Figure S1c presents the Raman characteristic peaks of monolayer WS_2_, where 349.8 cm^−1^ represents the in-plane vibrational peak and 419.2 cm^−1^ represents the out-of-plane vibrational peak of WS_2_ [27]. Photoluminescence (PL) testing of the WS_2_ showed a strong 632 nm PL peak, consistent with previous findings of monolayer WS_2_ [28]. Therefore, the uniformly distributed flakes on the SiO_2_/Si substrate were confirmed to be monolayer WS_2_.

Subsequently, to explore the fabrication conditions for two-dimensional Sb_2_Te_3_, mica substrates, which lack dangling bonds similar to monolayer WS_2_ surfaces, were utilized. Figure S2 shows optical images of two-dimensional Sb_2_Te_3_ at different positions on a 1.5 cm × 1.5 cm mica substrate. Figure S3a presents an optical image of thin-layer Sb_2_Te_3_ crystals, while Figure S3b shows an AFM image of Sb_2_Te_3_ with a measured height of 3.8 nm corresponding to four layers of Sb_2_Te_3_ [14]. Figure S3c shows the Raman spectrum of Sb_2_Te_3_ highlighting the characteristic Raman peaks A^1^_1g_ (69.8 cm^−1^), E^2^_g_ (112.1 cm^−1^), and A^2^_1g_ (164.9 cm^−1^) [29]. A low-resolution TEM image of two-dimensional Sb_2_Te_3_ is depicted in Figure S4a. Figure S4b illustrates a high-resolution image, with a 0.22 nm interplanar spacing, consistent with the reported (100) plane spacing of Sb_2_Te_3_ [14]. Figure S4c displays a selected-area electron diffraction pattern of two-dimensional Sb_2_Te_3_, consistent with the reported hexagonal pattern. Finally, energy-dispersive spectroscopy testing of the crystals, as shown in Figure S4e,f, revealed the uniform distribution of Sb and Te elements within the flake. Thus, the flakes formed on the mica substrate were identified as large-area, uniformly distributed two-dimensional Sb_2_Te_3_.

**Figure 1:**
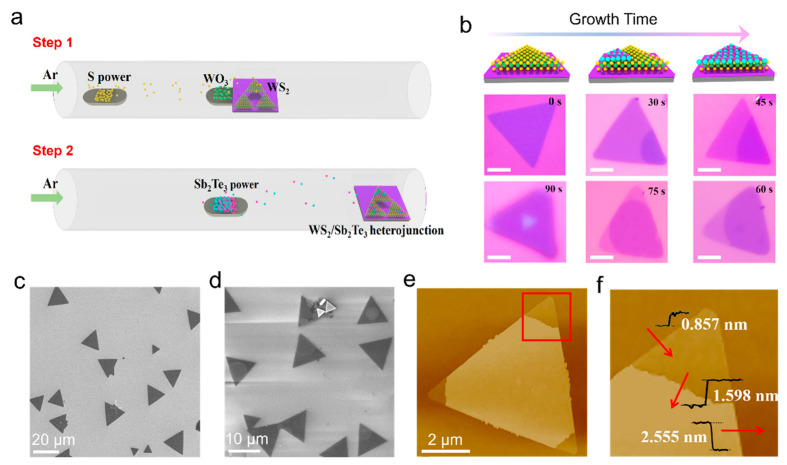
Synthesis of vertically stacked Sb_2_Te_3_/WS_2_ van der Waals heterojunctions: (**a**) schematic overview of the growth process for the synthesis of Sb_2_Te_3_/WS_2_ heterojunctions using a dual-stage chemical vapor deposition process; (**b**) schematic diagrams illustrating the growth process of the Sb_2_Te_3_/WS_2_ heterostructures over time, in which the deep purple in the same triangular flake represents the Sb_2_Te_3_/WS_2_ heterostructures, while the light purple represents monolayer WS_2_ (scale bar: 10 µm); (**c**) scanning electron microscope (SEM) image exhibiting a typical monolayer WS_2_ triangular flakes, while (**d**) exhibits the partially covered vertically stacked Sb_2_Te_3_/WS_2_ heterostructures; (**e**) Atomic Force Microscope (AFM) images of the partially covered Sb_2_Te_3_/WS_2_ heterojunction; (**f**) the magnified view of the red square area depicted in (**e**).

The preparation process for the Sb_2_Te_3_/WS_2_ vertical heterostructure involved substituting the mica substrate with a single layer of WS_2_ flakes grown on a SiO_2_/Si substrate. Figure 1a illustrates the schematic of the synthesis of Sb_2_Te_3_/WS_2_ vertical heterostructures utilizing a two-step chemical vapor deposition (CVD) method. During this process, Sb_2_Te_3_ powders were placed at the central end of the quartz reactor, while a SiO_2_/Si substrate with pre-fabricated WS_2_ flakes was positioned downstream. The thermal evaporation of Sb_2_Te_3_ powders enabled the vertical epitaxial growth of Sb_2_Te_3_ over the monolayer WS_2_ (detailed growth process described in Section 2.4*. Synthesis of 2D-Sb_2_Te_3_/WS_2_ Heterostructure*). The growth dynamics of Sb_2_Te_3_/WS_2_ vertical heterostructures were meticulously examined. As depicted in Figure 1b, Sb_2_Te_3_ selectively grew on the surface of WS_2_. The pronounced optical contrast vividly illustrates the morphological evolution of Sb_2_Te_3_/WS_2_ grains, progressing from incomplete to complete coverage with increasing growth time from 0 to 90 s. Representative SEM micrographs of pure WS_2_ samples and Sb_2_Te_3_/WS_2_ heterostructures are shown in Figure 1c and Figure 1d, respectively. The SEM micrograph depicts a typical vertically arranged Sb_2_Te_3_/WS_2_ heterojunction, with Sb_2_Te_3_ partially covering the underlying WS_2_ layer. Evidently, Sb_2_Te_3_ flakes tend to deposit on the WS_2_ surface, with minimal residual sediment observed on the SiO_2_/Si substrate. AFM was utilized for sample characterization, as illustrated in Figure 1e,f. The thickness measurements of WS_2_ and Sb_2_Te_3_ at 0.857 nm and 1.598 nm, respectively, confirm a monolayer structure for WS_2_ and a bilayer structure for Sb_2_Te_3_, termed as 2QL (with each quintuple layer (QL) in Sb_2_Te_3_ arranged in a Te-Sb-Te-Sb-Te sequence) [30,31]. Additionally, the analysis of the heterostructure morphology demonstrates the uniform surface quality of both Sb_2_Te_3_ and WS_2_.

**Figure 2:**
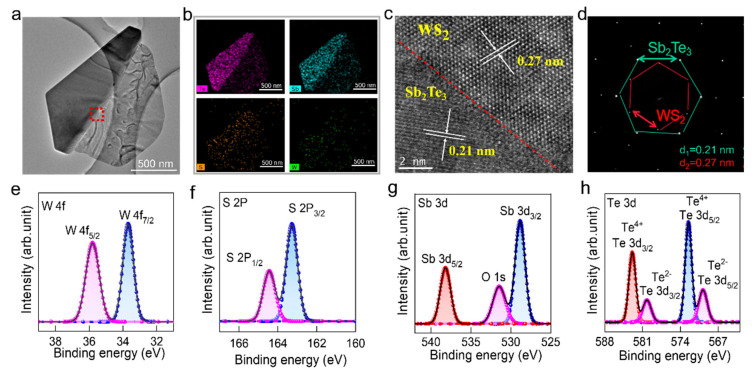
Atomic configuration of the vertically aligned Sb_2_Te_3_/WS_2_ heterojunction: (**a**) low-magnification TEM image of a vertically aligned Sb_2_Te_3_/WS_2_ vdW heterojunction, the red dashed rectangular region corresponds to the location in the high-resolution TEM image in (**c**); (**b**) a 2D elemental mapping visualizing the distribution of W, S, Sb, and Te within the Sb_2_Te_3_/WS_2_ heterostructure; (**c**) high-resolution TEM image capturing the interface region of the heterojunction; (**d**) electron diffraction pattern captured from the aligned region of the Sb_2_Te_3_/WS_2_ vdW heterojunction; (**e**–**h**) XPS spectra of W 4f, S 2p, Sb 3d, and Te 3d levels in the Sb_2_Te_3_/WS_2_ heterostructure, with black lines denoting measured data and dots representing fitting curves.

The atomic arrangement of the vertically stacked Sb_2_Te_3_/WS_2_ van der Waals heterojunction was further investigated using TEM coupled with energy-dispersive spectroscopy (EDS). Figure 2a displays a low-magnification TEM image of the as-transferred Sb_2_Te_3_/WS_2_ sample on a copper grid. Figure 2b presents an elemental distribution map of Te, Sb, S, and W originating from the marked sample region in Figure 2a. Notably, W and S elements exhibit uniform distribution throughout the entire area, whereas Sb and Te elements predominantly concentrate within the dark triangular region. Figure S5 presents the EDS spectra obtained from Figure 2a. The high-resolution TEM image in Figure 2c focuses on the region delineated by the red dashed line in Figure 2a. The contrasting regions reveal a lattice spacing of approximately 0.27 nm on the right, corresponding to the (100) plane of hexagonal WS_2_ [32], and a lattice spacing of 0.21 nm on the left, corresponding to the (100) plane of hexagonal Sb_2_Te_3_ [14]. Furthermore, the clarity within the heterojunction of lattice fringe patterns demonstrates its superior crystal quality. The selected area electron diffraction (SAED) pattern featured in Figure 2d provides compelling evidence of the crystallographic structure of the Sb_2_Te_3_/WS_2_ heterojunction. This pattern exhibits two distinct sets of diffraction patterns: one corresponding to the lattice of Sb_2_Te_3_, characterized by a spacing of 0.21 nm, and another corresponding to the lattice of WS_2_, characterized by a spacing of 0.27 nm. These distinct diffraction patterns unequivocally confirm the single-crystal nature of both Sb_2_Te_3_ and WS_2_, thereby underscoring the high-quality crystalline properties exhibited by the heterojunction.

X-ray photoelectron spectroscopy (XPS) analysis (shown in Figure 2e–h) was utilized as a tool for elucidating the chemical composition of the Sb_2_Te_3_/WS_2_ heterostructure. In Figure 2e, distinct peaks observed at binding energies of 33.7 and 35.8 eV correspond to the chemical states of W 4f_7/2_ and W 4f_5/2_, respectively. Additionally, peaks observed at binding energies of 163.4 and 164.5 eV are indicative of the chemical states of S 2p_3/2_ and S 2p_1/2_ in Figure 2f, respectively [33]. The XPS data for Sb 3d and Te 3d are presented in Figure 2g and Figure 2h, respectively. The fitted curves for Sb 3d at 528.8 and 538.2 eV correspond to Sb 3d_3/2_ and Sb 3d_5/2_ [34]. Meanwhile, Te 3d displays a peak at 569.7 eV for Te 3d_5/2_, and 580.1 eV for Te 3d_3/2_ [15,35]. Notably, higher binding energy peaks at 573.5 eV and 582.9 eV are attributed to tellurium oxide formation resulting from surface oxidation [36]. The values measured for S 2p, W 4f, Sb 3d, and Te 3d are consistent with reported values for WS_2_ and Sb_2_Te_3_. These analyses suggest that, during the top Sb_2_Te_3_ vapor growth process, no additional impurities infiltrate the underlying WS_2_ flake. Overall, the TEM and XPS investigations affirm the high crystal quality exhibited by the fabricated Sb_2_Te_3_/WS_2_ van der Waals heterostructure.

**Figure 3:**
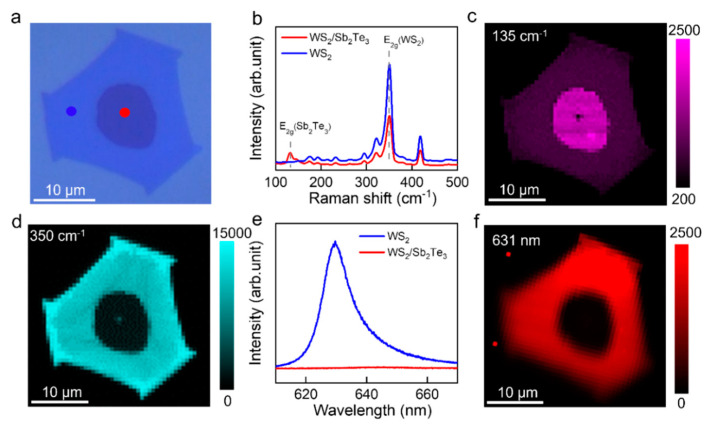
Utilization of 532 nm laser excitation for Raman and photoluminescence (PL) characterization of the Sb_2_Te_3_/WS_2_ heterojunction: (**a**) optical photograph of the Sb_2_Te_3_/WS_2_ heterojunction; (**b**) Raman spectra collected from the red dots (dark covered region at the crystal center) and blue dots (light region at the outer edge of the crystal) in (**a**); (**c**,**d**) frequency-specific Raman mapping of the Sb_2_Te_3_/WS_2_ heterojunction at 135 cm^−1^ and 350 cm^−1^, correspondingly; (**e**) photoluminescence spectra obtained from the isolated WS_2_ area (blue curve) and the intersected region (red curve); (**f**) PL cartography of the vertical stacked Sb_2_Te_3_/WS_2_ heterojunction, at 631 nm.

Raman and photoluminescence (PL) measurements were conducted utilizing a 532 nm laser excitation to comprehensively characterize the Sb_2_Te_3_/WS_2_ vertical heterojunction. Figure 3a exhibits the optical image of the partially covered Sb_2_Te_3_/WS_2_ heterojunction sample. Moving to Figure 3b, the Raman spectrum of the Sb_2_Te_3_/WS_2_ heterostructure reveals distinct Raman peaks corresponding to WS_2_ (E_2g_^1^ at 350 cm^−1^ and mode A_1g_ at 412 cm^−1^) [37] and Sb_2_Te_3_ (A_2u_^3^ at 135 cm^−1^) [34] within the junction region. This observation serves to validate the vertical configuration of the Sb_2_Te_3_/WS_2_ junction. Further examination through Raman mapping, as shown in Figure 3c,d, provides additional insights. The Raman mapping at 135 cm^−1^, shows uniform signal intensity distribution at the center of the crystal, significantly stronger than at the periphery, indicating uniform Sb_2_Te_3_ in the crystal center. Conversely, the Raman mapping at 350 cm^−1^ displays a uniform signal intensity distribution at the crystal’s periphery, stronger than at the center, signifying uniform WS_2_ at the crystal’s outer region. Notably, the stronger signal of WS_2_ at 350 cm^−1^, combined with the central Raman spectrum analysis, confirms a vertically stacked Sb_2_Te_3_/WS_2_ structure within the central circle region. Room temperature PL spectra and mapping are presented in Figure 3e,f. The PL mapping depicts strong PL emission at 631 nm from the single WS_2_ region (blue line in Figure 3e), while the PL of WS_2_ is significantly quenched in the vertically stacked Sb_2_Te_3_/WS_2_ heterostructure region (red line in Figure 3e). Normalization of the peak intensity of the PL spectra, as illustrated in Figure S6, unveils a redshift in the PL peak of the Sb_2_Te_3_/WS_2_ heterostructure. This redshift is attributed to strong charge transfer between WS_2_ and Sb_2_Te_3_, serving as the primary cause of the significant PL quenching and redshift in the PL peak position [38]. In summary, the successful fabrication of the Sb_2_Te_3_/WS_2_ vertical heterostructure is confirmed through comprehensive Raman and PL characterizations.

### 3.2. Density Functional Theory Calculations of Band Structures for Monolayer (1L) WS_2_ and Two-Dimensional (2D) Sb_2_Te_3_

**Figure 4:**
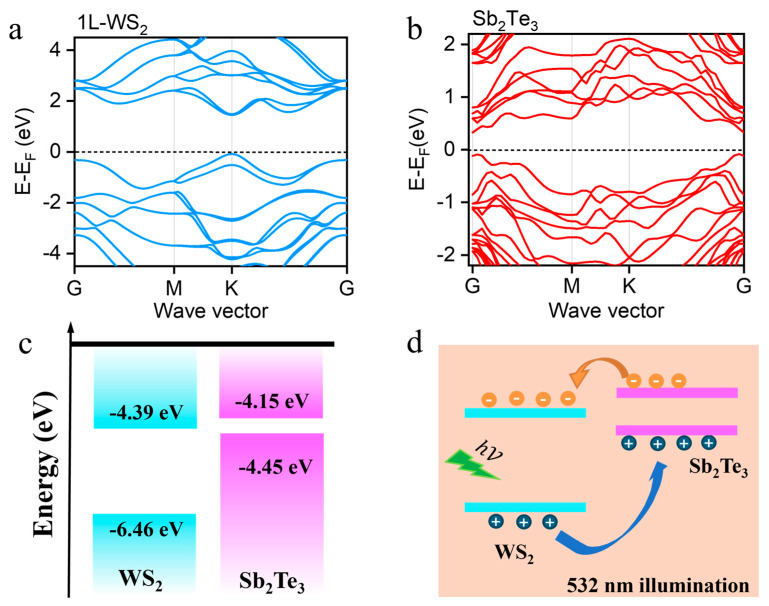
Schematic diagram of theoretical calculations: (**a**,**b**) calculated band structures of 1L WS_2_ and two-dimensional (2D) Sb_2_Te_3_; (**c**) energy band profiles of the 1L WS_2_ and Sb_2_Te_3_ before contract; (**d**) band alignment illustration displaying the process of charge transfer at the interface of the junction under 532 nm laser exposure.

Using density functional theory calculations, the band structure of monolayer WS_2_ and the band structure of 2D Sb_2_Te_3_ were individually calculated based on their respective structures, as illustrated in Figure 4a,b. According to existing reports, the conduction band minimum (CBM) and valence band maximum (VBM) of WS*_2_* are, respectively, at −4.39 eV and −6.46 eV [39]; whereas for Sb, the CBM and VBM are located at −4.15 eV and −4.45 eV [40], as demonstrated in Figure 4c. Upon the formation of heterostructures, a type-II band alignment is observed at the junction interface. Upon laser excitation at 532 nm, photoexcited electrons in Sb_2_Te_3_ tend to transfer to WS_2_, while holes in WS_2_ preferentially migrate to Sb_2_Te_3_. This charge separation mechanism hinders recombination within the heterostructure, leading to notable photoluminescence (PL) quenching (Figure 4d) and redshift in the PL peak position (Figure S6), consistent with the observed PL test results [38,39,40,41].

### 3.3. Optoelectronic Testing of Sb_2_Te_3_/WS_2_

**Figure 5:**
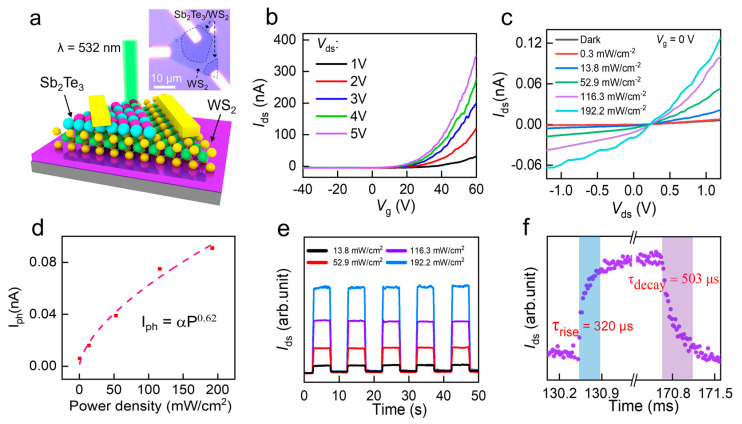
Optoelectronic performances of 2D Sb_2_Te_3_/WS_2_-based photodetectors: (**a**) schematic representation of the Sb_2_Te_3_/WS_2_-based photodetector—inset: optical image of the Sb_2_Te_3_/WS_2_ device; (**b**) transfer characteristics of the Sb_2_Te_3_/WS_2_ device in the absence of illumination; (**c**) I_ds_-V_ds_ curves of the Sb_2_Te_3_/WS_2_ device with 532 nm laser with power ranging from 0 to 192.18 mW/cm^2^; (**d**) relationship between photocurrent (I_ph_) and illumination intensity levels; (**e**) response of photocurrent in the p–n junction with light cyclically switched on and off under 532 nm laser illumination; (**f**) Time-evolved photoresponse of the p–n diode, specifically illustrating the photocurrent rise and decay duration.

The optoelectronic performance of the Sb_2_Te_3_/WS_2_ nanoflakes was investigated by fabricating Sb_2_Te_3_/WS_2_ based photodetectors on a SiO_2_/Si substrate, as illustrated in Figure 5a. Employing a Cr/Au electrode, one end was connected to the upper Sb_2_Te_3_ layer, while the other was linked to the lower WS_2_ layer. Figure 5b demonstrates the transfer characteristic curves of the Sb_2_Te_3_/WS_2_ p–n heterojunction. The prevailing n-type transfer curve suggests that electron transport dominates the charge transport within WS_2_. Figure 5c presents the I_ds_-V_ds_ curves of the Sb_2_Te_3_/WS_2_ p–n heterojunction under dark conditions and various power levels of 532 nm laser irradiation. With increasing optical power, the photogenerated current increases. Moreover, the power-dependent photoresponse was modeled using the power law ($I_{ph}=\alpha P^{\theta }$) to examine the trap states within the Sb_2_Te_3_/WS_2_ nanoflakes [42,43], as depicted in Figure 5d. The fitting coefficient of 0.62 implies that as laser intensity rises, light absorption gradually saturates. To assess the photoresponse speed of the Sb_2_Te_3_/WS_2_ heterojunction photodetector, time-resolved photoresponse measurements were conducted, with results depicted in Figure 5e. The photocurrent demonstrates efficient switching between on and off states by periodic activation and deactivation of the laser, showcasing exceptional stability. Figure 5f shows the rise/decay time of the photocurrent in the photodetector, where the rise/decay time is defined as the time required for the photocurrent to increase from 10% to 90% of the peak value and decrease from 90% to 10% of the peak value, respectively. Further analysis reveals a rise time (τ_rise_) of 305 μs and a decay time (τ_decay_) of 503 μs for the device, surpassing those of the WSe_2_/WS_2_ vertical heterostructure [44]. Given the excellent mechanical flexibility [25] and high integration of two-dimensional layered materials, their potential applications in future industrial flexible robotic sensors warrant additional research into two-dimensional optoelectronic devices.

## 4. Conclusions

In summary, we achieved a successful synthesis of large-sized two-dimensional Sb_2_Te_3_ on mica substrates via chemical vapor deposition. Through subsequent advancements, we attained precise deposition of Sb_2_Te_3_ onto the WS_2_ surface, resulting in the successful fabrication of Sb_2_Te_3_/WS_2_ heterojunctions. Fabricated photodetectors based on Sb_2_Te_3_/WS_2_ showed rapid response times, with a rise time of 305 μs and a decay time of 503 μs. This progress promotes the construction of heterojunctions between two-dimensional Sb_2_Te_3_ and various bandgap layered materials. Such progress not only paves the way for the realization of high-performance photodetectors but also propels the application of two-dimensional Sb_2_Te_3_-related heterojunctions across diverse fields.

## Data Availability

The data presented in this study are available upon request to the corresponding authors.

## Supplementary Materials

The following supporting information can be downloaded at: https://www.mdpi.com/article/10.3390/nano14100884/s1, Figure S1: Optical Microscope (OM), AFM, Raman, PL characterizations of monolayer WS_2_. Figure S2: large-area, uniformly distributed 2D Sb_2_Te_3_ obtained at different spots on the whole 1.5 cm × 1.5 cm mica substrate. Figure S3: AFM and Raman characterizations of 2D Sb_2_Te_3_. Figure S4: TEM, SAED, EDS characterizations of 2D Sb_2_Te_3_. Figure S5: detailed EDS spectrum of Sb_2_Te_3_/WS_2_ heterostructure. Figure S6: Peak intensity normalization performed on the PL spectra shown in Figure 3e.
