# Supplementary material for: Direct Selective Epitaxy of 2D Sb2Te3 onto Monolayer WS2 for Vertical p–n Heterojunction Photodetectors
Source: Nanomaterials (Basel). 2024 May 19;14(10):884. doi: 10.3390/nano14100884 (PMC11124104; doi:10.3390/nano14100884)
Supplement: Supplementary file 1 [file nanomaterials-14-00884-s001.zip › nanomaterials-2970970-supplementary.pdf]

Supplementary Information for

Direct Selective Epitaxy of 2D Sb_2Te_3 onto Monolayer WS_2 for Vertical p–n Heterojunction Photodetectors

Baojun Pan [1,†], Zhenjun Dou [2,†], Mingming Su [1], Ya Li [1], Jialing Wu [1], Wanwan Chang [1], Peijian Wang [2], Lijie Zhang [2], Lei Zhao [3,*], Mei Zhao [2,*] and Sui-Dong Wang [1]

[1] Macao Institute of Materials Science and Engineering (MIMSE), MUST-SUDA Joint Research Center for Advanced Functional Materials, Macau University of Science and Technology, Taipa, Macao 999078, China

[2] Key Laboratory of Carbon Materials of Zhejiang Province, Institute of New Materials & Industry Technology, College of Chemistry and Materials Engineering, Wenzhou University, Wenzhou 325035, China

[3] School of Electronic Engineering, Lanzhou City University, Lanzhou 730070, China

* Correspondence: zhaolei@lzcu.edu.cn (L.Z.); zhaomei@wzu.edu.cn (M.Z.)

† These authors contributed equally to this work.

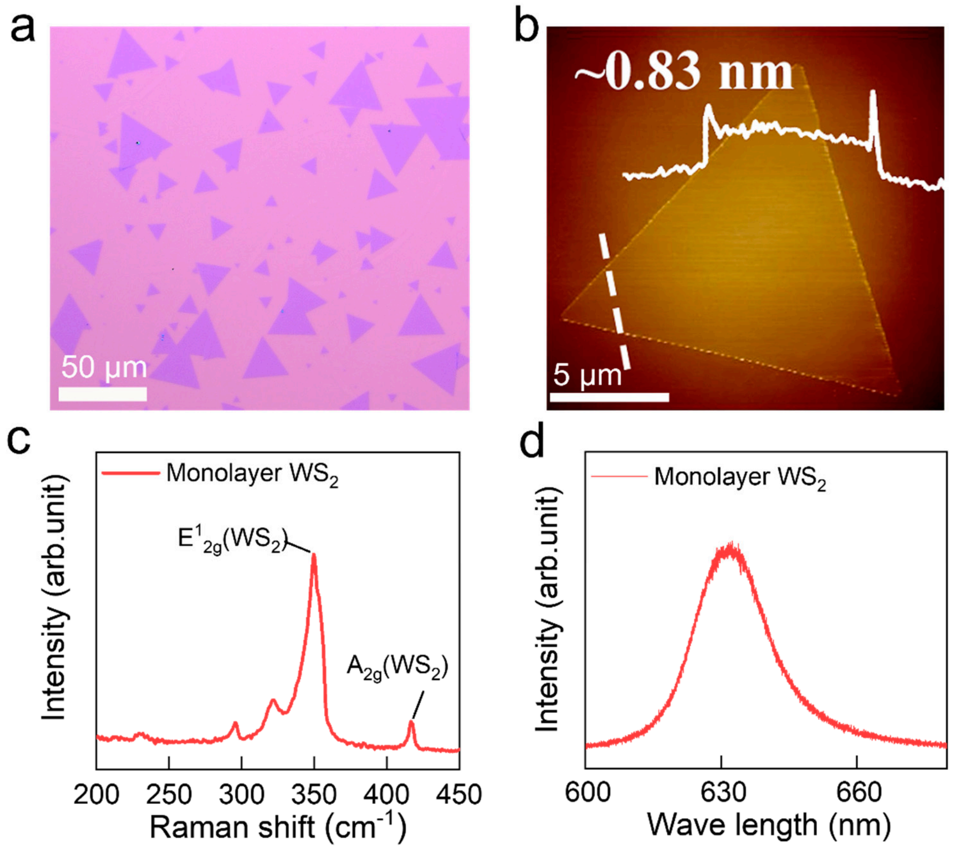

Figure S1. (**a**) The optical image of large-area, uniform monolayer WS_2 flakes. (**b**) AFM images of the WS_2. (**c**) The Raman spectroscopy of the monolayer WS_2 flake. (**d**) Photoluminescence (PL) spectroscopy of the monolayer WS_2 flake.

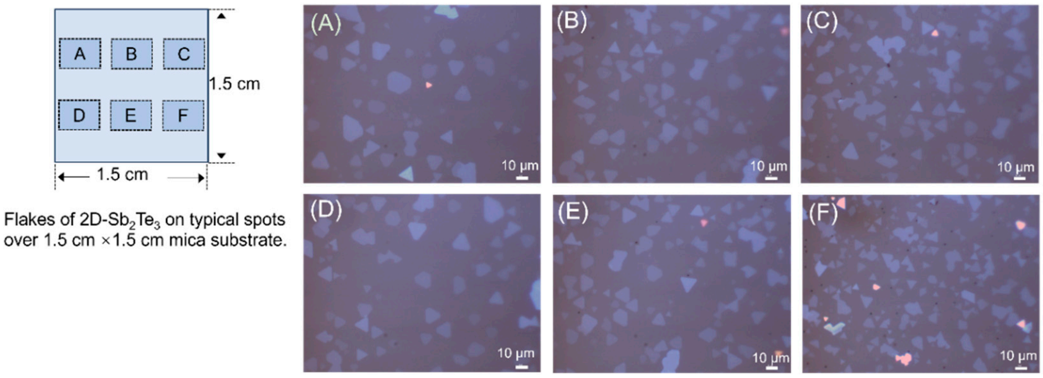

Figure S2. Large-area, uniformly distributed two dimension Sb_2Te_3 obtained at different spots on the whole 1.5 cm×1.5 cm mica substrate, corresponding to the schematics in the left side.

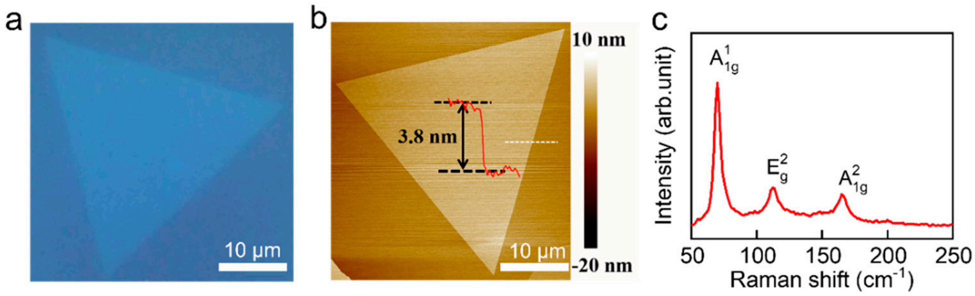

Figure S3. AFM and Raman characterizations of two-dimensional Sb_2Te_3. (**a**) Optical image of two-dimensional Sb_2Te_3. (**b**) Atomic force microscopy image of two-dimensional Sb_2Te_3. (**c**) Raman spectrum of two-dimensional Sb_2Te_3.

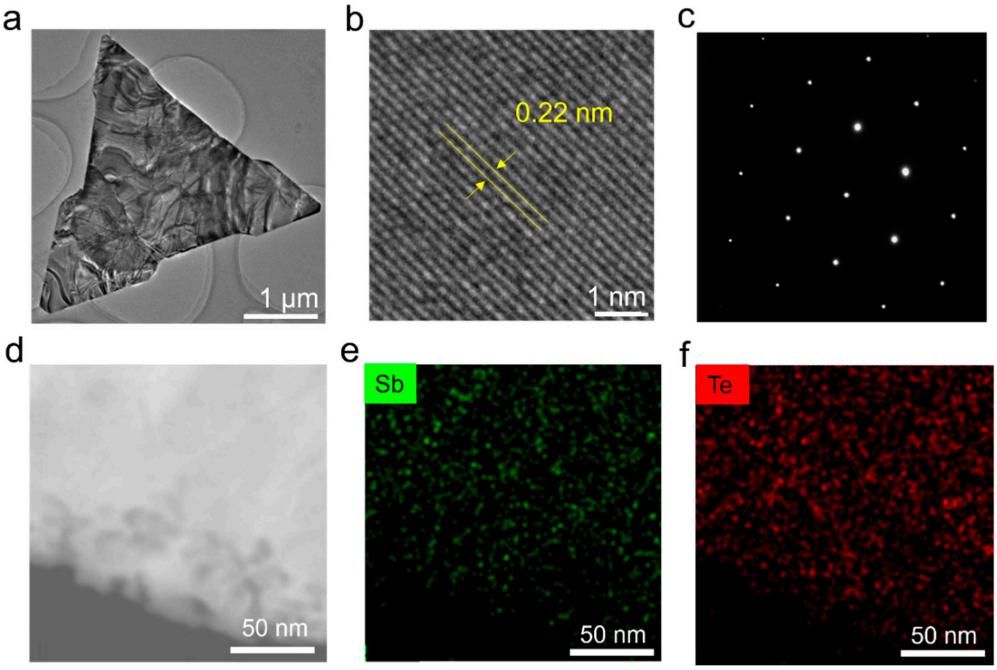

Figure S4. Atomic structure of the two dimension Sb_2Te_3. (**a**) Low-magnification TEM image of two dimension Sb_2Te_3. (**b**) High-resolution TEM image of the Sb_2Te_3. (**c**) Electron diffraction pattern taken on the stacked region of the two dimension Sb_2Te_3. (**d**–**f**) Collecting EDS mapping of the area and the distribution maps of: Sb and Te, respectively.

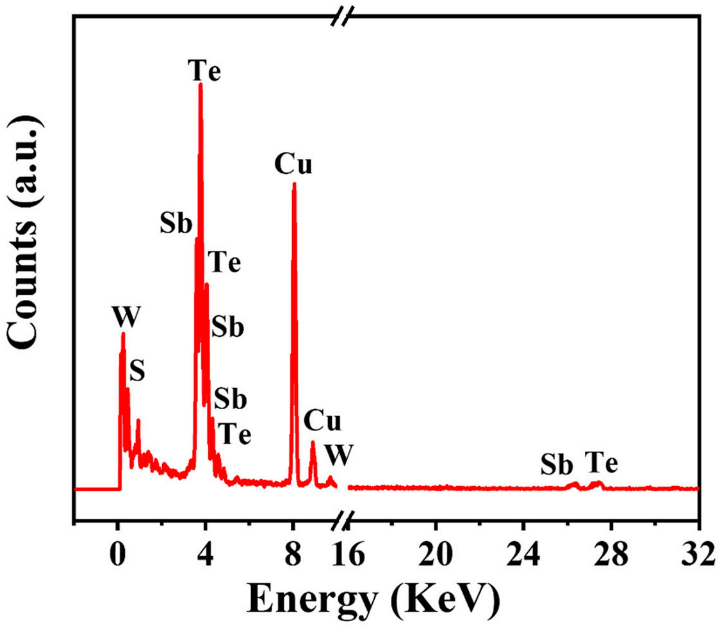

Figure S5. Detailed EDS spectrum of Sb_2Te_3/WS_2 heterostructure.

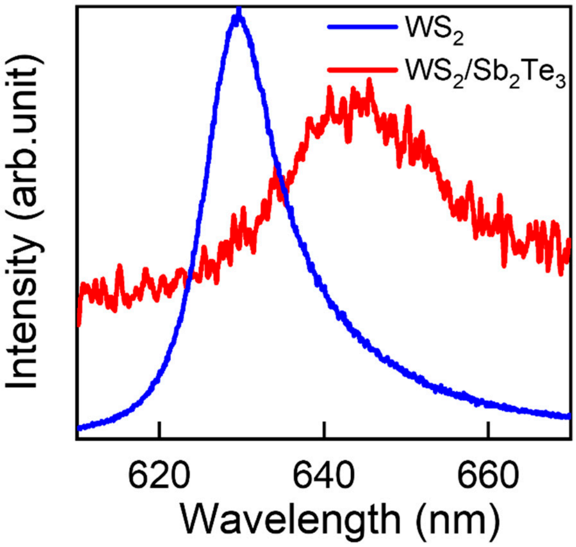

Figure S6. Peak intensity normalization performed on the PL spectra shown in Figure 3e.
